# Thermal analysis of a reflection mirror by fluid and solid heat transfer method

**DOI:** 10.1107/S1600577524008749

**Published:** 2024-10-15

**Authors:** Zhen Wang, Fang Liu, Chaofan Xue

**Affiliations:** a Center for Transformative Science ShanghaiTech University 393 Middle Huaxia Road Shanghai201210 People’s Republic of China; b Shanghai Institute of Applied Physics Chinese Academy of Sciences Shanghai201204 People’s Republic of China; Tohoku University, Japan

**Keywords:** finite-element analysis, water cooling, thermal analysis, heat flux, heat transfer

## Abstract

High-repetition-rate free-electron lasers impose stringent requirements on the thermal deformation of beamline optics. The Shanghai HIgh-repetition-rate XFEL aNd Extreme light facility (SHINE) experiences high average thermal power and demands wavefront preservation. To deeply study the thermal field of the first reflection mirror M1 at the FEL-II beamline of SHINE, thermal analysis under a photon energy of 400 eV was executed by fluid and solid heat transfer method. According to the thermal analysis results and the reference cooling water temperature of 30 °C, the temperature of the cooling water at the flow outlet is raised by 0.15 °C, and the wall temperature of the cooling tube increases by a maximum of 0.5 °C. The maximum temperature position of the footprint centerline in the meridian direction deviates away from the central position, and this asymmetrical temperature distribution will directly affect the thermal deformation of the mirror and indirectly affect the focus spot of the beam at the sample.

## Abstract 2

By thermal analysis of the reflection mirror in a free-electron laser under high repetition rates within the fluid and solid heat transfer method, the water temperature increase of the cooling tube was found to influence the wall temperature of the fluid–solid interface and results in an asymmetrical temperature distribution of the footprint centreline in the mirror. Here, a more accurate thermal analysis of the reflection mirror under a high heat load is presented that will improve the cooling scheme design of the mirror.

## 1. Introduction

**Figure 1:**
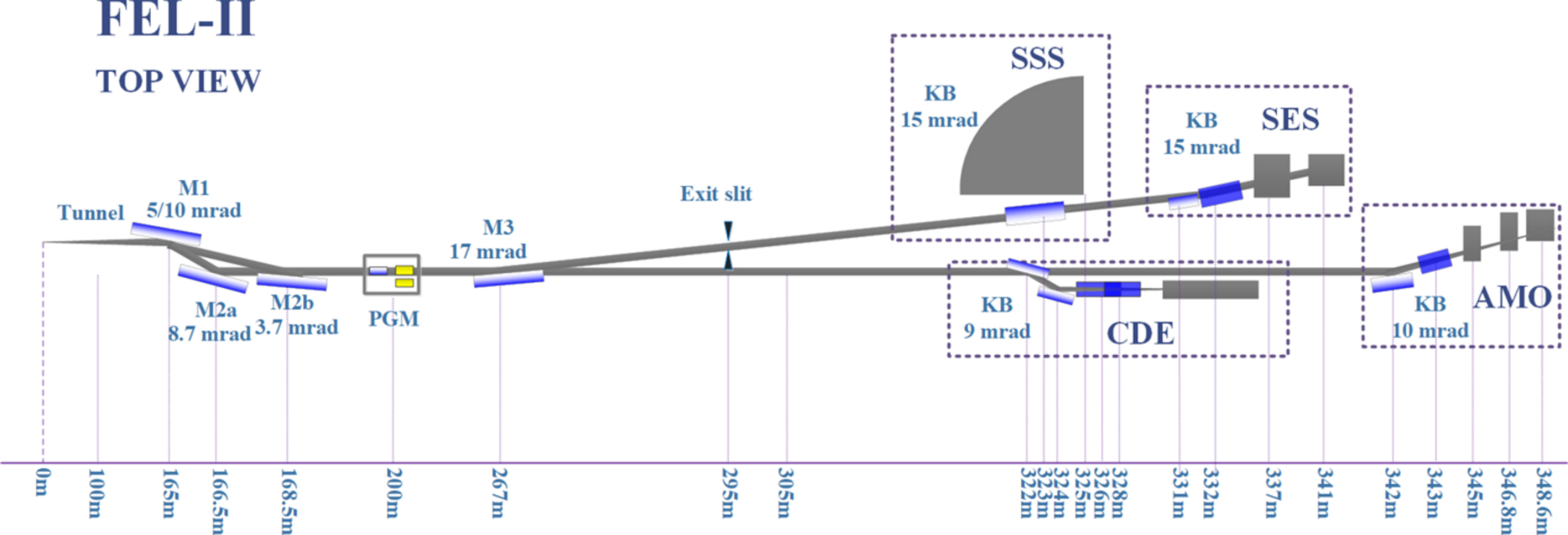
Optical path of beamline FEL-II at SHINE.

The SHINE facility, located in Shanghai, is a remarkable achievement as it stands as China’s first high-repetition-rate X-ray free-electron laser (XFEL) device. In the first phase, three beamlines were built, FEL-I, FEL-II and FEL-III, that covered the photon energy range between 0.4 and 25 keV. The FEL-II beamline operates within the photon energy range 0.4–3 keV and contains four experimental stations: Soft X-ray Scattering Spectrometer endstation (SSS), Spectrometer for Electronic Structure endstation (SES), Coherent Diffraction Endstation (CDE) and Atomic, Molecular, and Optical Physics endstation (AMO) (Liu *et al.*, 2023 ▸), as displayed in Fig. 1 ▸.

XFELs, renowned for their high brightness and coherence (Kim *et al.*, 2017 ▸), have revolutionized scientific research with their ultra-short pulses, evolving from traditional X-ray tubes to diffraction-limited storage rings and XFEL sources (Eriksson *et al.*, 2014 ▸; Hettel, 2014 ▸; Emma *et al.*, 2010 ▸; Ishikawa *et al.*, 2012 ▸). The new generation of high-repetition-rate XFELs presents unique challenges (Cocco & Spiga, 2019 ▸; Church & Takacs, 1993 ▸), such as high heat loads on optical components like the first mirror (M1), which can lead to thermal deformation and impact imaging quality. Project SHINE addresses these challenges, focusing on thermal management to preserve wavefront integrity and ensure optimal imaging performance.

Water-cooling is widely used to address the high heat load of optical components in synchrotron radiation and FEL facilities. Traditionally, to simulate the water-cooling, the equivalent convective heat transfer coefficient with a constant temperature has been applied (Signorato *et al.*, 1998 ▸; Yang *et al.*, 2014 ▸; Sutter *et al.*, 2022 ▸; Zhang *et al.*, 2013 ▸). However, this method ignored the increase of water temperature along the flow direction during the cooling process of the cooling water, as well as the temperature change of the fluid–solid interface caused by the rise in water temperature and the influence of the temperature distribution in the footprint of the mirror.

This paper presents thermal analysis for a high-heat-load mirror by the fluid and solid heat transfer method, in which the increase of cooling water temperature, temperature change of the fluid–solid interface, and temperature distribution of the centerline in the footprint were studied. The mirror assembly is first introduced, then the finite-element model of *Multiphysics* analysis is explained, and a more accurate thermal field of the mirror under the high heat load is analyzed. A more realistic thermal analysis of the mirror will be more beneficial for the design of cooling schemes.

## 2. Finite-element method

Thermal analysis of a high-heat-load mirror was carried out by the coupled model of the Nonisothermal Flow and Conjugate Heat Transfer in COMSOL *Multiphysics* (https://www.comsol.com), which calculates the heat transfer in a solid, across a solid–fluid interface, and in a non-isothermal cooling fluid (John *et al.*, 2019 ▸).

### 2.1. Geometric model

**Figure 2:**
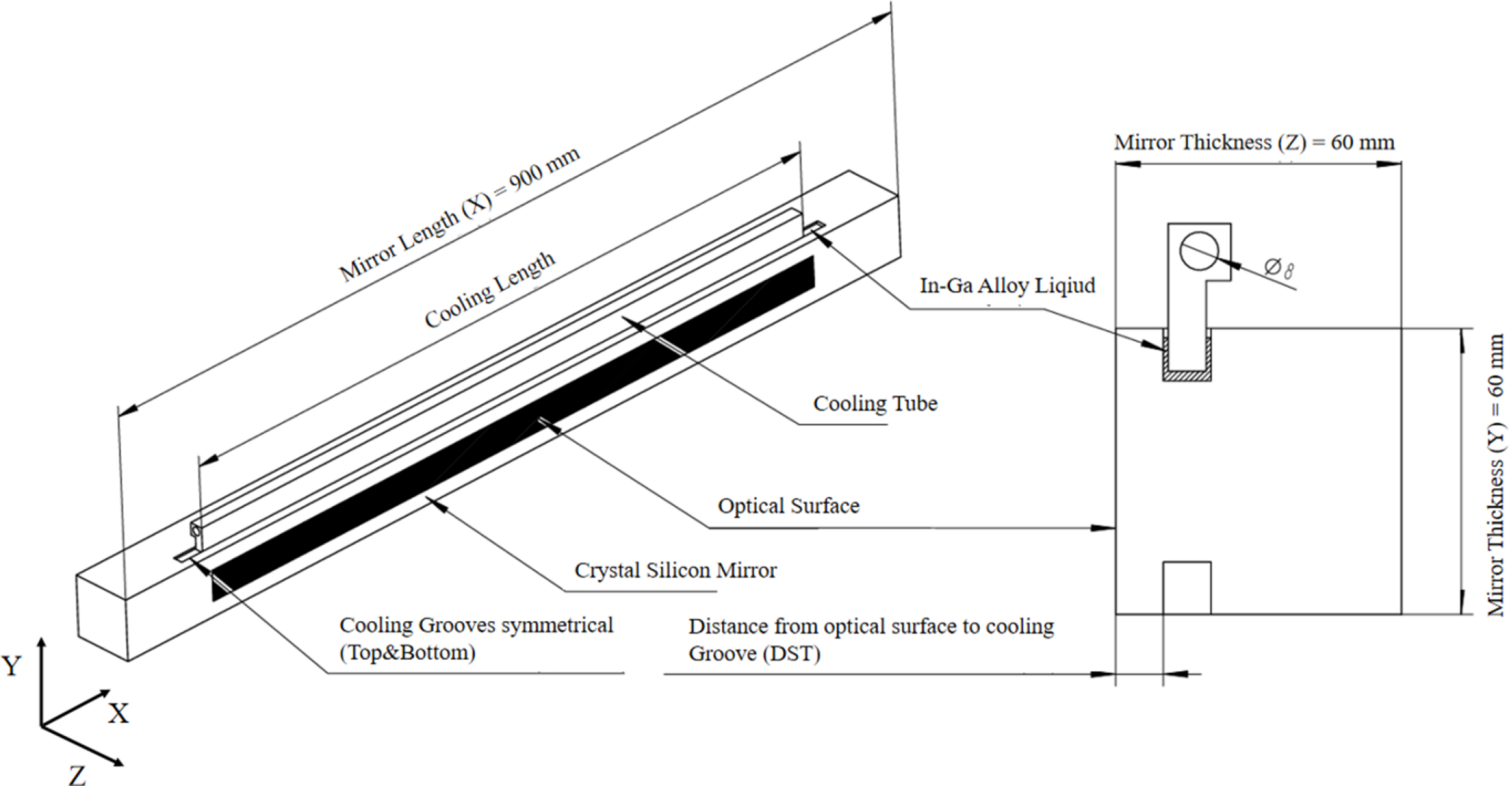
Layout of the mirror with cooling blade.

According to the current optical design requirements for the FEL-II beam, the mirror M1 was designed as shown in Fig. 2 ▸. It was determined that the mirror size should be 900 mm (*x*) × 60 mm (*y*) × 60 mm (*z*), and made from single-crystal silicon due to its exceptional thermal conductivity and low thermal expansion coefficient. Owing to the significant heat load issue, the cooling blade for the mirror has been designed and fabricated using oxygen-free copper material to ensure optimum heat transfer. The cooling blade tube featured an inner diameter of 8 mm, further enhancing the heat transfer capability of the mirror system. To ensure the stability standards of the 3.1 km hard X-ray free-electron laser facility, it is recommended to consider the application of liquid indium–gallium eutectic in the upper cooling groove of the mirror.

Table 1 ▸ and Table 2 ▸ list the materials utilized based on the FEL-II beam design specifications for Project SHINE.

### 2.2. Heat load and boundary conditions

**Figure 3:**
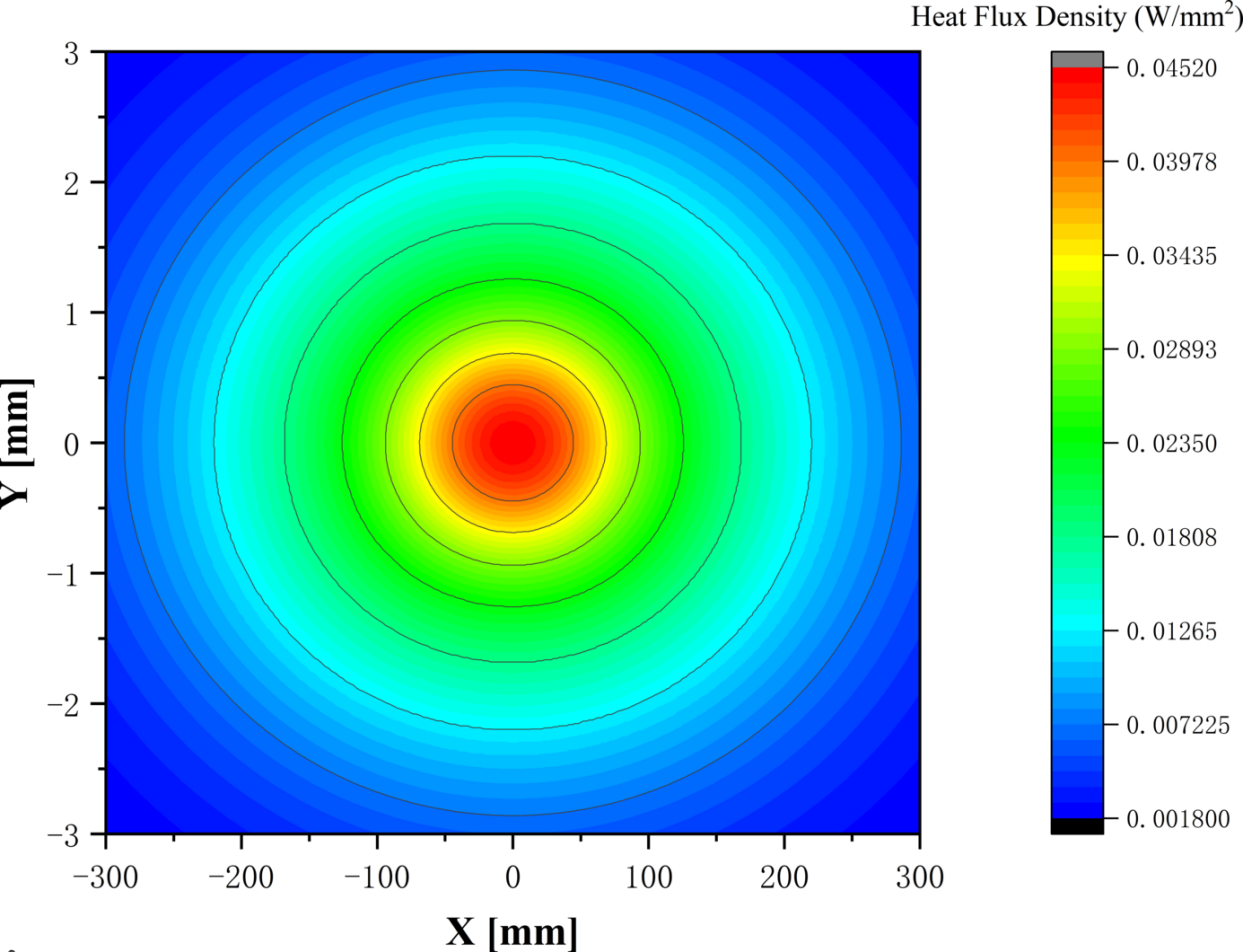
Heat load distribution on reflection mirror M1 for a photon energy of 400 eV.

The mirror was located 165 m from the light source, and the incident angles on the first reflection mirror’s optical surface were set at 5 mrad for photon energy points of 2000 eV and 3000 eV, and increased to 10 mrad specifically for the lower photon energy points of 200 eV, 400 eV, 900 eV and 2000 eV. For the study case of 400 eV photon energy, the footprint size on the optical surface was 600 mm (*X*) × 6 mm (*Y*), and the heat load was focused on the center of the mirror’s optical surface. Fig. 3 ▸ displays the heat flux density distribution of the footprint zone. Mirror M1 at a repetition rate of 1 MHz absorbed a maximum heat load of 48.6 W under a photon energy of 400 eV, resulting in a peak power density of 0.0452 W mm^−2^.

Moreover, the contact conductance coefficient between the indium–gallium alloy and the single-crystal silicon in the mirror system and cooling tube was 150000 W m^−2^ K^−1^ (Khounsary *et al.*, 1997 ▸). The cooling medium with an initial flow rate of 1.5 L min^−1^ in the cooling tube was applied at a temperature of 30 °C.

### 2.3. Finite-element model

**Figure 4:**
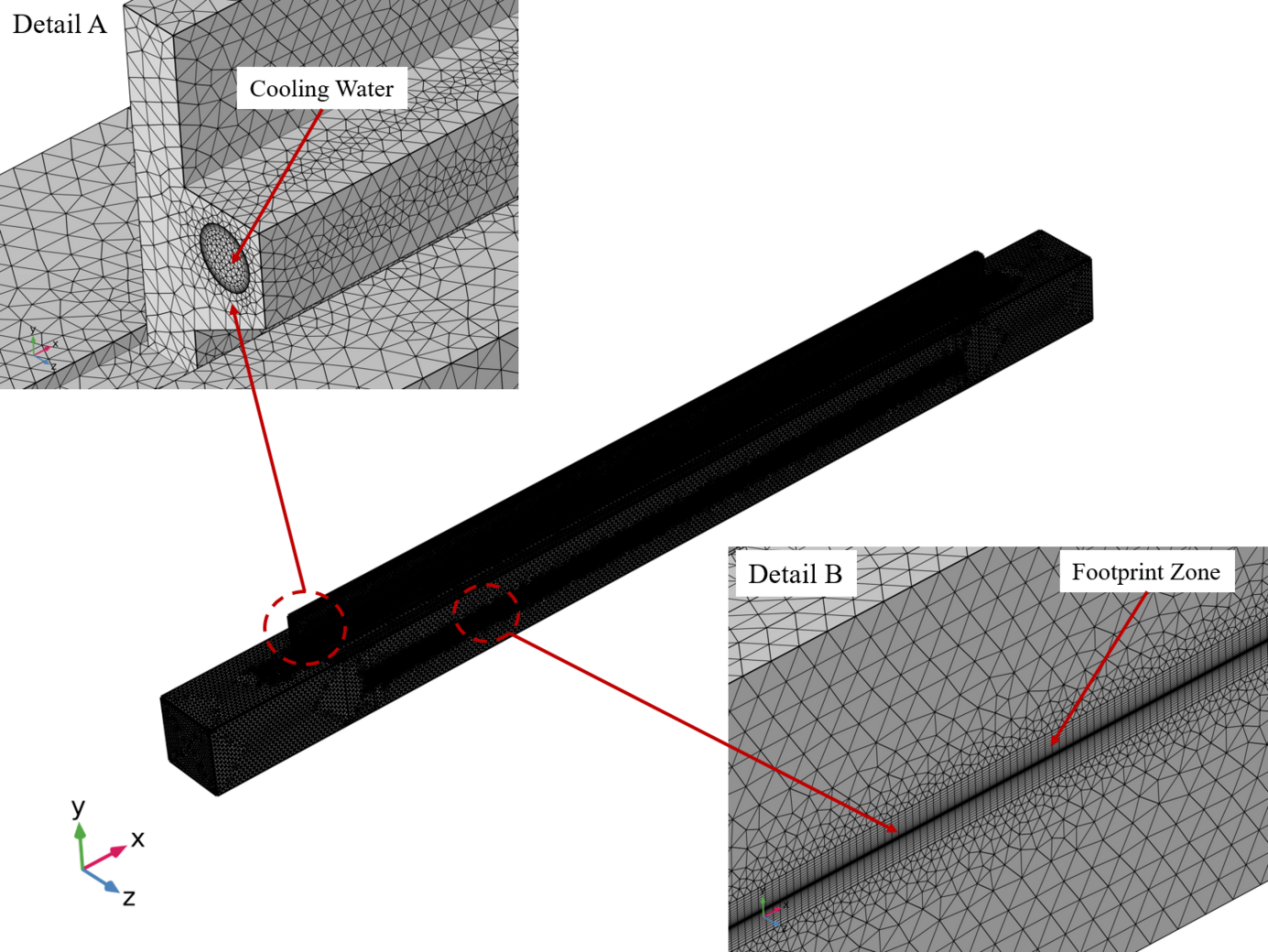
Finite-element model of the mirror system. Detail A displays the mesh of the solid–fluid interface zone. Detail B shows the mesh of the footprint area.

The mirror system assembly’s finite-element analysis (FEA) model, displayed in Fig. 4 ▸, utilized a global element size of 4 mm and tetrahedral element type. A local fine mesh was employed to account for the Gaussian distribution characteristics of heat density in the central region with high thermal density, with some areas reaching a footprint of 0.25 mm. The entire finite-element model was made up of 2990674 elements and 724233 nodes.

## 3. Results and discussion

The thermal analysis of the mirror within the non-isothermal flow *Multiphysics* coupling was conducted to simulate fluid flows and solid heat transfer, performed under a 330 kHz repetition rate to satisfy the experimental station’s specifications for the focused beam spot. Within the analysis results, a detailed study was performed, focusing on the temperature distribution and the heat flux flow of the mirror assembly, and the variations in temperature in both the water and at the fluid–solid interface, and a description of the beam’s focus spot on the sample. The above studies will provide a deeper understanding of the thermal field of high-heat-load mirrors within a cooling system.

### 3.1. Temperature results of the *Multiphysics* analysis

**Figure 5:**
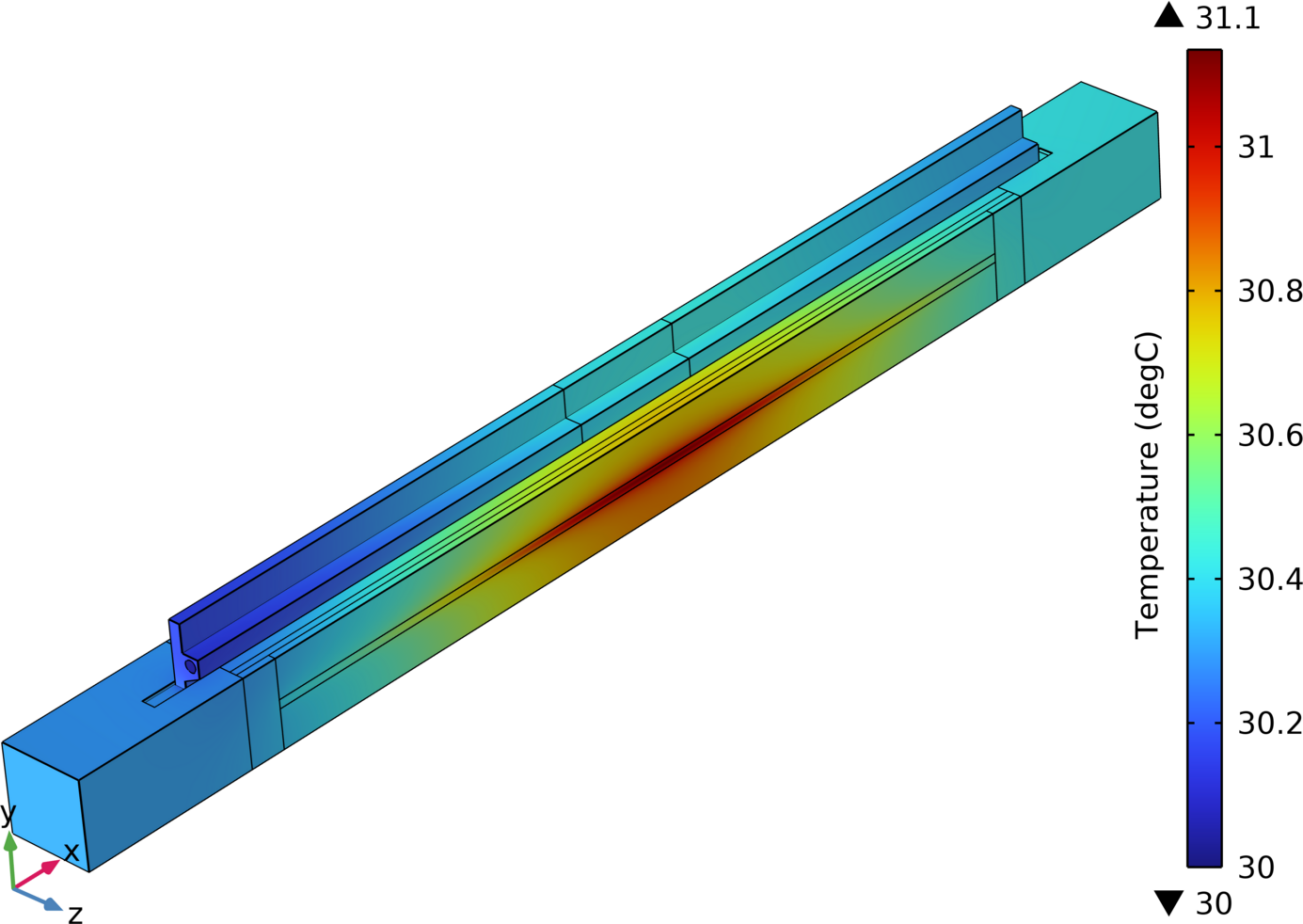
Temperature distribution of the reflection mirror assembly.

The results of the *Multiphysics* simulation showed that the highest temperature occurred in the center of the footprint, with a maximum of 31.1 °C, and the lowest temperature of 30 °C is displayed in the flow inlet position of the cooling blade, as shown in Fig. 5 ▸. In addition, it can be seen from Fig. 5 ▸ that the temperature field across the entire analytical model is asymmetrically distributed in the mirror length direction, and the temperature of the left side of the analysis model is significantly lower than that of the right side. The main reason is that the cooling water flows in the +*X* direction from the flow inlet to the flow outlet and is constantly heated by the heat load of the reflection mirror, which leads to a rise in the water temperature in the flow direction and so further reduces the cooling effects of the cooling water on the right side of the mirror model.

### 3.2. Heat flux flow of the mirror assembly

**Figure 6:**
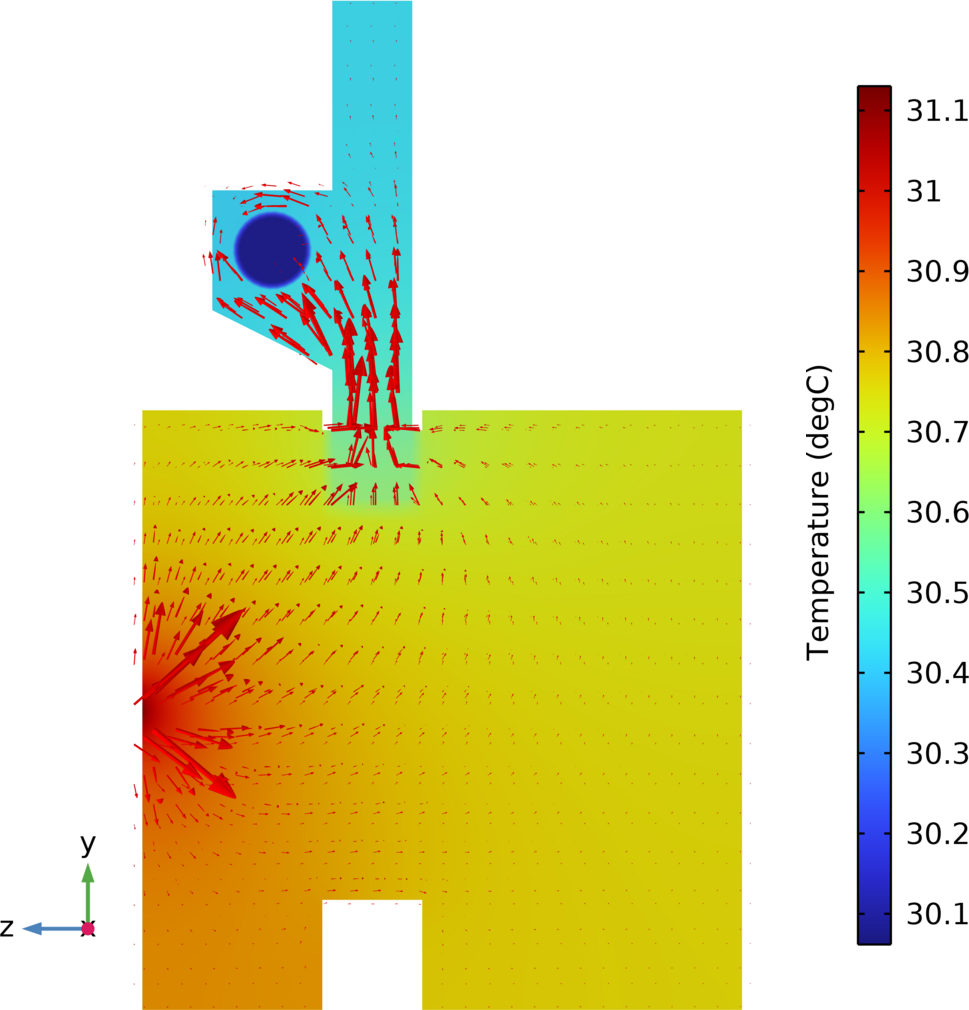
Temperature distribution and heat flux flow at the *YZ* plane and *x* = 0 mm.

Heat flux flow describes the flow of heat during thermal analysis and can be used to describe the transfer of heat through a region or system. Fig. 6 ▸ shows the heat flow path of the analysis model in a view of a defined finite-element model cross-section, where the heat flow is first radially transferred from the footprint position of the mirror to the periphery. Under the effect of the temperature difference between the mirror and the cooling water (the highest temperature occurred at the mirror footprint and the lowest temperature located at the water of the cooling blade), heat flows to the cooling blade through the intermediate medium (liquid indium–gallium) and finally to the cooling tube.

**Figure 7:**
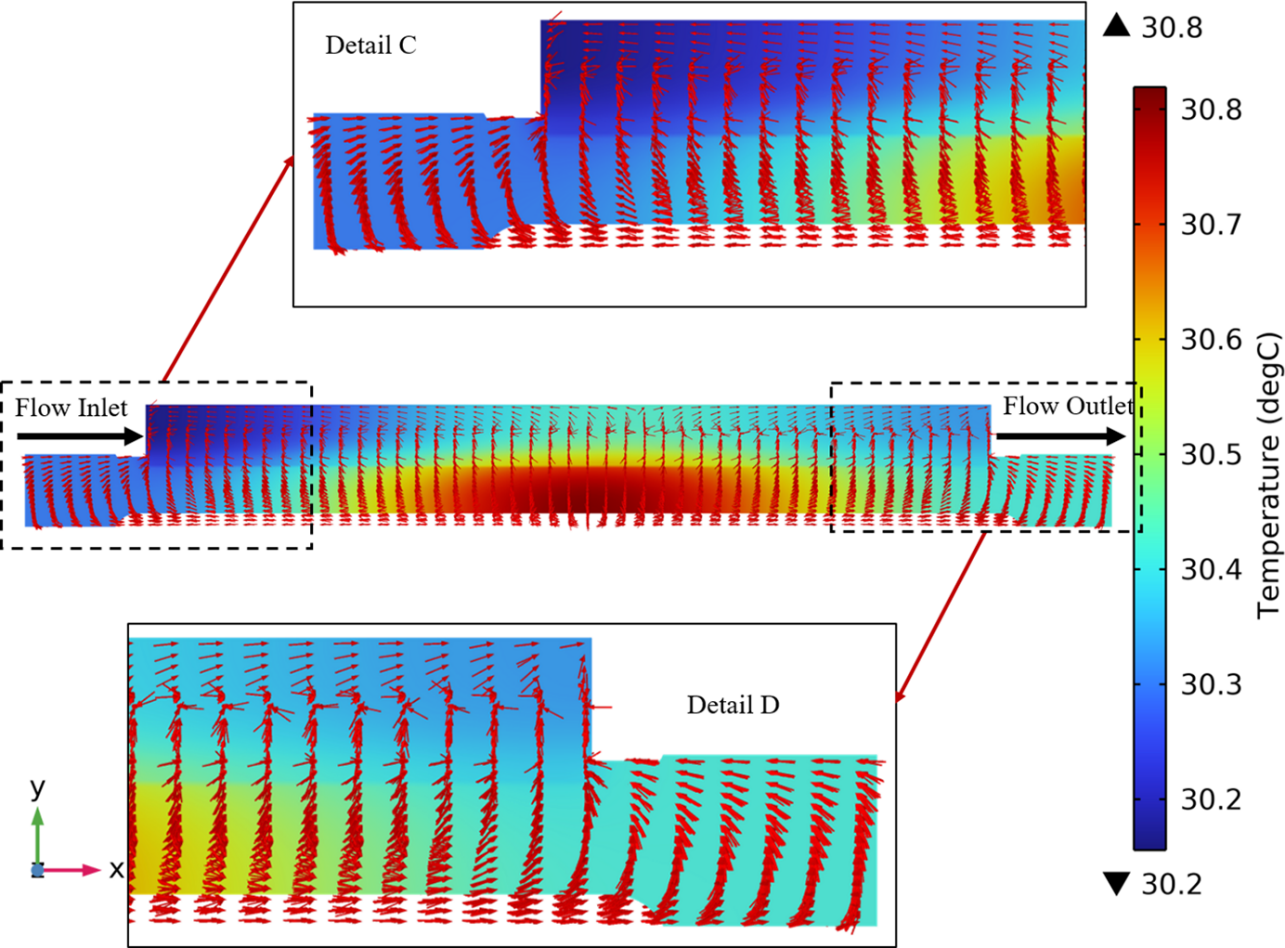
Temperature distribution and heat flux flow at the *XY* plane and *z* = −23 mm.

Similarly, at the top position of the cooling blade in Fig. 7 ▸, more heat flux can be seen flowing along the length direction of the mirror to the left half of the cooling blade. This phenomenon shows that the temperature of the left half of the cooling blade is relatively low, and the temperature of the cooling water gradually increases with the direction of the water flow while effectively dissipating the heat of the mirror.

As depicted in Detail C and Detail D of Fig. 7 ▸, in the length direction of the mirror, owing to the good thermal conductivity of the mirror and the significant temperature difference of the mirror assembly, the heat flow is conducted radially from the center of the mirror to other positions and the cooling blade. In particular, the heat flow at both ends of the mirror reversely flowed to the cooling blade with a lower temperature.

### 3.3. Temperature variation of water, wall and footprint centreline along the meridian direction

**Figure 8:**
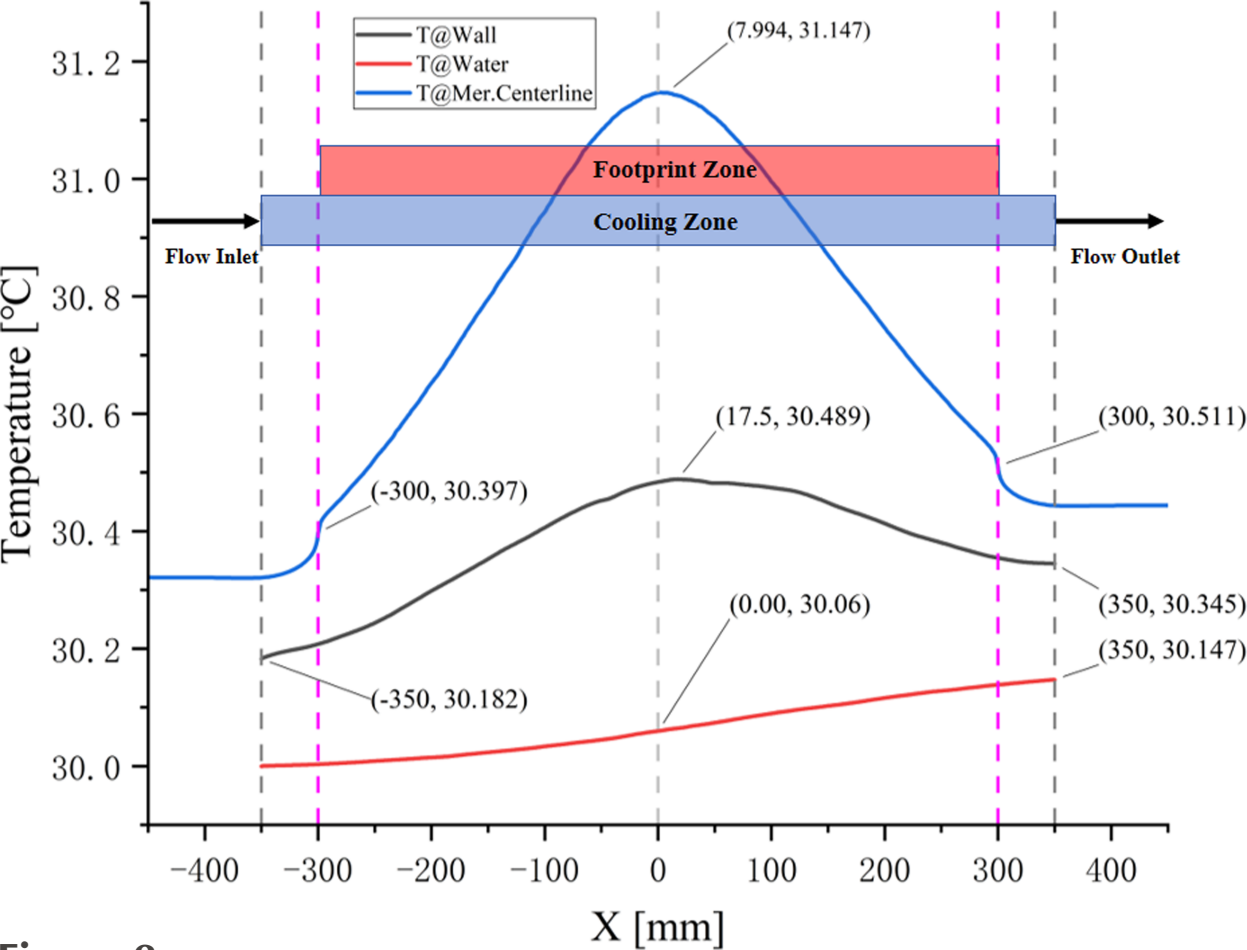
Temperature change of the water, wall and meridian centreline of the footprint in the mirror model. Note that the water temperature is measured at the center point of the cooling tube cross-section; the wall temperature is presented as the temperature of the fluid–solid interface. Mer. Centerline indicates the centreline of the footprint in the meridian direction.

The variation trends of the water temperature, wall temperature and the temperature of the footprint centreline are presented in Fig. 8 ▸. While the cooling water dissipated the heat load of the mirror, the internal energy of the cooling water (entropy) increased, leading to a rise in the water temperature along the flow direction. Although the water temperature difference between the flow inlet of 30 °C and the flow outlet of 30.147 °C reached only 0.147 °C, it still influenced the temperature distribution of the mirror assembly.

Firstly, due to the continuous increase of the cooling water temperature, the highest temperature position of the footprint centerline in the mirror is +8 mm away from the center position (*X* = 0 mm). Similarly, the highest temperature position of the fluid–solid interface occurs at +17.5 mm from the center point. The position of the highest temperature will cause the maximum thermal deformation of the mirror to deviate from the center of the footprint.

Secondly, the temperature difference between the two ends of the cooling pipe wall increased by 0.163 °C, from 30.182 °C at the water inlet to 30.345 °C at the water outlet, and the maximum temperature of the wall reached 30.489 °C at the position *X* = +17.5 mm, which is nearly 0.5 °C higher than the water reference temperature of 30 °C.

Finally, as shown in Fig. 8 ▸, the temperature of the footprint centerline in the meridian direction is asymmetrical, mainly due to the increase in water temperature, which led to asymmetrical thermal distortion that directly affects the position of the beam focus point on the sample.

### 3.4. Discussion

**Figure 9:**
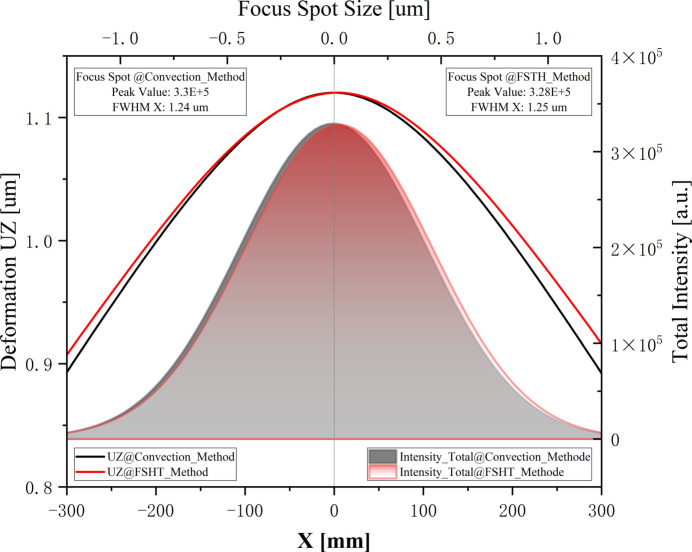
Comparison of thermal deformation and focused spots on the sample position obtained by the two above-mentioned methods. Note that, to better contrast the thermal deformations obtained by the two calculation methods, we overlap their thermal deformation at position *X* = 0, so that the difference between the two thermal deformations is even more obvious.

In the traditional method, to simulate the water-cooling effect of high-heat-load mirrors, a fixed convective heat transfer coefficient is applied on the cooling tube wall. By this convection method the temperature distribution and thermal deformation in the meridian direction on the footprint will be symmetrical. Compared with the convection method, as depicted in Fig. 9 ▸, the fluids and solids heat transfer (FSHT) method delivered a relatively larger and asymmetrical thermal deformation, which is more realistic. Additionally, this asymmetrical thermal deformation in the +*X* direction is significantly larger than that in the −*X* direction. This was attributed to the method’s consideration of the increased cooling water temperature along the +*X* direction, enhancing the thermal analysis accuracy of the high-heat-load mirrors.

The thermal deformation of the mirror will directly impact the quality of the beam’s focus spot at the sample, including aspects such as the intensity of the focal spot, size of the focus spot, position of the focal spot, *etc*. By conducting optical tracing based on the mutual optical intensity (Meng *et al.*, 2015 ▸) with the thermal deformations obtained from the two aforementioned simulation methods, the intensity distribution curve of the beam’s focus spot on the sample was determined and is shown in Fig. 9 ▸. It was evident that the beam’s focus spot derived from an asymmetric thermal deformation experienced a smaller overall shift relative to that from a symmetric thermal deformation. Concurrently, there was a reduction in intensity of the beam’s focus spot, which had declined from 3.3 × 10^5^ to 3.28 × 10^5^. Additionally, the half width at half-maximum of the focal spot has undergone a slight variation, increasing from 1.24 µm to 1.25 µm.

Therefore, this advanced approach can provide a relatively more realistic thermal field for high-heat-load mirrors and will improve thermal management techniques for high-heat-load optics of X-ray beamlines.

## 4. Conclusion

This *Multiphysics* analysis presented the thermal field of high-heat-load mirrors under cooling, with a focus on temperature distribution, heat flux flow, and temperature variations across the mirror assembly. Advanced numerical simulations have demonstrated the complex interplay of heat transfer between the cooling water and the reflection mirror, enhancing the understanding of thermal management in high-repetition-rate optics of X-rays.

The numerical results indicated that the temperature distribution of the reflection mirror along the mirror length direction is asymmetric, due to the influence of the rise in the cooling water temperature along the flow direction. This asymmetry directly affects the transmission of the beam and the quality of the beam’s focus spot on the sample, and is a necessary consideration of thermal deformation management for high-heat-load mirrors.

In conclusion, departing from traditional equivalent film coefficient methods, this coupled fluid and solid heat transfer approach can provide a more accurate thermal analysis result of high-heat-load mirrors. It is significant for designing and optimizing cooling systems for high-heat-load mirrors of X-rays.

*Note added in proof.* We would like to extend our special thanks to Dr Howard Padmore (Advanced Light Source, Lawrence Berkeley National Laboratory, and Physics Department, Arizona State University, USA) for his theoretical validation of the water temperature rise in the thermal analysis section of this article.

## Figures and Tables

**Table 1:** Properties of the applied materials (Wang *et al.*, 2022 ▸)

Material	Density (kg m^−3^)	Elastic modulus (GPa)	Poisson ratio	Yield stress (MPa)	Thermal conductivity (W m^−1^ °C^−1^)	Thermal expansion coefficient (°C^−1^, 25°C)
Silicon	2329	112.40	0.28	120.00	148.00	2.50 × 10^−6^
Copper	8900	110.00	0.34	220.00	391.00	1.75 × 10^−5^
In–Ga	6350	–	–	–	28	–

**Table 2:** Properties of the applied cooling medium water (Xu *et al.*, 2024 ▸)

Material	Density (kg m^−3^)	Thermal conductivity (W m^−1^ K^−1^)	Specific heat (J kg^−1^ K^−1^)	Viscosity (mPa s)
H_2_O	998.19	0.598	4179	1.0016

## Data Availability

Data will be made available on request.
